# Significance of red cell distribution width measurement for the patients with isolated coronary artery ectasia

**DOI:** 10.1186/1479-5876-12-62

**Published:** 2014-03-07

**Authors:** Xiao-Lin Li, Li-Feng Hong, Yan-Jun Jia, Shao-Ping Nie, Yuan-Lin Guo, Rui-Xia Xu, Cheng-Gang Zhu, Li-Xin Jiang, Jian-Jun Li

**Affiliations:** 1 Division of Dyslipidemia, State Key Laboratory of Cardiovascular Disease, Fu Wai Hospital, National Center for Cardiovascular Disease, Chinese Academy of Medical Sciences and Peking Union Medical College, Beijing 100037, China; 2 Department of Cardiology, Fifth Hospital of Wuhan City, Wuhan 430050, China; 3 Department of Cardiology, Beijing Anzhen Hospital, Capital University of Medical Sciences, Beijing 100029, China

**Keywords:** Red cell distribution width, Coronary artery ectasia, Coronary artery disease, C-reactive protein

## Abstract

**Background:**

Red cell distribution width (RDW) has been recognized as a novel marker for several cardiovascular diseases. The aim of this study was to evaluate the association between RDW levels and the presence of isolated coronary artery ectasia (CAE).

**Methods:**

We studied 414 subjects including 113 patients with isolated CAE (Group A), 144 patients with coronary artery disease (CAD, group B) and 157 angiographically normal controls (group C). Baseline clinical characteristics and laboratory findings including RDW were compared among three groups.

**Results:**

The levels of RDW were significantly higher in group A and B compared with that in group C (12.97 ± 1.4 and 12.88 ± 1.0 vs 12.34 ± 0.9, p = 0.020) while no difference was found between CAE and CAD (p = 0.17). Additionally, the levels of CRP were also higher in patients with CAE and CAD compared with normal controls (0.26 ± 0.14 mg/L, 0.31 ± 0.27 mg/L vs 0.20 ± 0.06 mg/L, p = 0.04). The multivariate analysis indicated that RDW and CRP were the independent variables most strongly associated with the presence of isolated CAE and CAD. There was a positive correlation between levels of RDW and CRP in patients with isolated CAE (γ=0.532, p = 0.001).

**Conclusions:**

Our data suggested that RDW may be a useful marker and independent predictor for the presence of isolated CAE.

## Introduction

Coronary artery ectasia (CAE) is a common finding of coronary angiography, which is characterized by abnormal coronary dilatation and defined as dilated coronary artery segments that are greater than 1.5 times the diameter of adjacent normal segment [1,2]. Although previous studies have demonstrated that CAE could predispose to adverse coronary events like vasospasm, thrombosis, dissection, and even myocardial infarction [3-5], the underlying mechanisms for this unique vascular disease are still unclear.

Previous investigation indicated the atherosclerotic lesion might be a potential cause for the development of CAE because it was frequently coincident with coronary artery disease (CAD) in some patients [4,5]. However, a few observations have also suggested that CAE could be found in a number of patients independent from the apparent atherosclerotic stenosis, called as the isolated CAE [4]. Therefore, exploration the potential biomarkers to discrimination isolated CAE from CAD may be important for clinical implication.

The red blood cell distribution width (RWD), part of a routine complete blood count, is a measure of the variability in the size of circulating erythrocytes and it has been utilized in the differential diagnosis of anemia [6]. Recently, a lot of previous studies have linked the baseline RDW to predicting the presence and outcomes of several cardiovascular diseases including acute coronary syndrome, stable angina, heart failure, peripheral vascular disease, stroke, and thrombosis after percutaneous coronary intervention due to acute myocardial infarction, cardiac syndrome X, even slow coronary flow syndrome [6-14].

Based on the above evidence, we hereby evaluate the association between RDW levels and the presence of CAE using the patients with isolated CAE as a study model.

## Methods

### Study population

The study population consisted of 414 patients including 113 patients with isolated CAE (group A) and 144 patients with CAD (group B) and 157 angiographically normal controls (group C) who underwent coronary angiography in our centers between January 2010 and December 2012 for a variety of indications. The study population was selected in a consecutive manner. The protocol was approved by Fu Wai hospital ethics committee, and complied with the Declaration of Helsinki.

CAE was defined as coronary arteries with a luminal dilatation of 1.5 fold or more of the adjacent normal coronary segment without significant coronary stenosis in this study according to previously reported investigations [1,2]. If there was no adjacent segment, mean diameters of the control patients were used for the related segment [1]. Patient with CAE but no significant obstructive coronary artery disease (less than 30% coronary stenosis) was defined as the isolated CAE including localized/focal or diffuse dilatation of a coronary artery [1].

CAD was defined as the left main coronary artery (LM), the left anterior descending artery (LAD), the left circumflex coronary artery (LCX), right coronary artery (RCA) or the main branch of the vascular diameter stenosis reaching 50% or more. The patients with significantly concomitant CAD (more than 30% stenosis in any coronary arteries) were excluded. The normal controls were defined as (1) the presence of anginal chest pain, (2) a normal coronary angiography, and (3) no ischemia on myocardial perfusion scintigraphy or during the treadmill exercise test.

All subjects enrolled in this study had normal hepatic and renal function. The hyperlipidemia was defined as low-density lipoprotein cholesterol 160 mg/dl^3^ and/or triglyceride (TG) 200 mg/dl^3^. Patients with history or evidence of CAD, valvular heart disease, congestive heart failure, left ventricular dysfunction, echocardiographically proven left ventricular hypertrophy, a history of dysphagia, swallowing as well as intestinal motility disorders, untreated thyroid disease, sinus node dysfunction or conduction disturbance, estrogen replacement therapy, carcinoma, poorly controlled hypertension (systolic blood pressure >160 mmHg or diastolic blood pressure >105 mmHg), recent major operation (<3 months), autoimmune disease were also excluded from the study.

In addition, patients who have had previous history of anemia, have received previous red blood cell transfusion or were on treatment for anemia, such as supplemental iron, folate or an erythropoiesis-stimulating agent were not included in this study. Patients with known hematological disease such as hemolytic anemia, neoplastic metastases to the bone marrow, iron replacement therapy that could increase plasma RDW levels were excluded.

### Coronary angiography

Left ventricular and selective coronary angiography for all enrolled individuals was performed using the standard Judkin’s techniques, and the results were analyzed by at least two interventional physicians according to our previous study [4].

### Laboratory examinations

EDTA-anticoagulated peripheral blood sample were taken from all enrolled patients after 12-hour overnight fast for the measurement of laboratory parameters. Hemoglobin, RDW and white blood cell (WBC) counts were determined by the automated hematology analyzer XE-1200 (Sysmex, Kobe, Japan). The normal range of RDW (%) in our laboratory was 10-16%. The other biochemical measurements were performed using a molecular analyzer (Roche Diagnostics, Manheim, Germany).

The plasma was obtained after a centrifugation of 3000 rpm at 4°C for 15 minutes. The levels of high-sensitivity C-reactive protein (hs-CRP) were determined using immunoturbidometry (Beckmann Assay 360, Bera, Calif., USA) as our previously reported [4]. The median normal value for CRP is 0.8 mg/L, with 90% of normal values <0.3 mg/L, with a lower detection limit of 0.2 mg/L. The inter- and intra-assay coefficients of variation were 4.4% and 3.5% respectively.

### Statistical analysis

Data were analyzed using SPSS statistical software, version 11.5 (SPSS Inc., Chicago, IL, USA). Continuous variables were expressed as mean±SD, and categorical variables were expressed as percentage. Comparison of categorical and continuous variables between the groups was performed using chi-square test and ANOVA test, respectively.

Because the distribution of hs-CRP was skewed rightward, log transformation was made at baseline, and the significance of any difference in distributions was assessed by the Wilcoxon rank-sum test as our previously reported [4]. The univariate and multivariate analysis was used for baseline clinical characteristics, hs-CRP and RDW. In a receiver operating characteristic (ROC) curve analysis, an RDW value of was identified as an effective cut-point in the segregation of the presence or absence of CAE. Finally, association between RDW and hs-CRP was test using Spearman correlation coefficient. A p value <0.05 was considered statistically significant.

## Results

### Baseline clinical characteristics

Baseline clinical characteristics of the population studied with isolated CAE (n = 113), CAD (n = 144) and normal controls (n = 157) are presented in Table 1. There were no significant differences among the groups with respect to body mass index, family history of CAD, function of left ventricle, and the percentage of lipid profile, diabetes. However, compared with group B and group C, the patients with isolated CAE were younger and were predominantly male. Additionally, the percentage of smoking and hypertension was significantly higher in patients with CAE group compared with control group.

**Table 1:** Baseline clinical characteristics of study population

**Variables**	**CAE group**	**CAD group**	**Control group**	**p value**
	**(n = 113)**	**(n = 144)**	**(n = 157)**	
Age (years)	48±10	57±18	54±13	0.036
Sex (F/M)	97/16	110/34	121/36	0.044
BMI (kg/m^2^)	24±4	24±3	24±4	0.748
Family history of CAD, n (%)	16 (14)	31 (22)	30 (19)	0.270
Current smoker, n (%)	48 (43)	32 (22)	15 (10)	0.041
Hypertension, n (%)	34 (31)	39 (27)	31 (20)	0.035
Hyperlipidemia, n (%)	31 (27)	40 (28)	53 (34)	0.509
Diabetes, n (%)	13 (12)	22 (15)	20 (13)	0.713
LVEF (%)	58±6	61±8	60±7	0.824
Medications on admission				
Aspirin, n (%)	52 (46)	60 (42)	58 (37)	0.192
β-blocke, n (%)	25 (22)	30 (21)	29 (19)	0.307
ACEI/ARB, n (%)	22 (20)	33 (23)	27 (17)	0.316
Statin, n (%)	17 (15)	22 (16)	19 (12)	0.818
CCB, n (%)	9 (8)	14 (10)	14 (9)	0.934

Data are presented as frequencies (percentages) for categorical variables and means±SD. CAE = coronary artery ectasia; CAD = coronary artery disease; BMI = body mass index; CAD = family history of CAD; LVEF = left ventricular ejection fraction; ACEI/ARB = angiotensin-converting enzyme inhibito/angiotensin receptor blocker; CCB = calcium channel blocker.

### Biomarker for predicting CAE

Laboratory findings from individuals with isolated CAE and those with CAD and normal controls are summarized in Table 2. As showed in Table 2, there were no differences among the groups regarding erythrocyte count, hemoglobin, mean corpuscular volume, and creatinine levels. However, The levels of RDW were significantly higher in group A and B compared with that in group C (12.97 ± 1.4 and 12.88 ± 1.0 vs 12.34 ± 0.9, p = 0.020) while no difference was found between CAE and CAD (12.97 ± 2.4 vs.12.88 ± 2.0, p = 0.17). In addition, the levels of CRP were also higher in patients with CAE and CAD compared with normal controls (0.26 ± 0.14 mg/L, 0.31 ± 0.27 mg/L vs 0.20 ± 0.06 mg/L, p = 0.04). Moreover, The data also showed an increased numbers of white blood cells in patients with isolated CAE and CAD group compared with normal controls (6347±1114/mm^3^ and 6109±1037/mm^3^ vs 5833±972/mm^3^, p = 0.038). Similarly, the peripheral circulating monocyte cells were significantly higher in patients with CAE group compared with CAD and normal controls (620±187/mm^3^ vs 553±170/mm^3^ and 521±107/mm^3^, p = 0.043).

**Table 2:** Laboratory findings of the study population

**Variables**	**CAE group**	**CAD group**	**Control group**	**p value**
	**(n = 113)**	**(n = 144)**	**(n = 157)**	
WBC (/mm^3^)	6347±1114	6109±1037	5833±972	0.038
PMC (/mm^3^)	620±187	553±170	521±107	0.043
Erythrocyte (10^12^/L)	4.2±0.4	4.3±0.5	4.3±0.3	0.677
HB (g/dl)	12.9±1.3	13.1±1.5	13.6±0.7	0.823
MCV (fL)	83±4.7	85±4.9	83±4.9	0.912
Creatinine (mg/dl)	0.84±0.52	0.88±0.57	0.88±0.93	0.844
hs-CRP (mg/L)	0.26±0.14	0.31±0.27	0.20±0.06	0.042
RDW (%)	12.97±1.4	12.88±1.0	12.34±0.9	0.020

Data are presented as means±SD. CAD = coronary artery disease; WBC = white blood cell; PMC = peripheral monocyte count; HB = Hemoglobin; Mean corpuscular volume Hs-CRP = high-sensitivity C-reactive protein; RDW = red blood cell distribution width.

### Independent risks for predicting CAE by multivariate analysis

Al total of 7 variables associated with isolated CAE including age, sex, smoking, peripheral circulating white blood cell, moncyte cell, hs-CRP and RDW with p < 0.05 in univariate analysis are presented in Table 3. And then, we put those 7 variables into the multivariate analysis. Interestingly, we found that sex, smoking, hs-CRP and RDW were the independent variables most strongly associated with isolated CAE (sex: odds ratio 1.13, 95% confidence interval 1.13 to 2.25, p = 0.006; smoking: odds ratio 1.22, 95% confidence interval 1.08 to 2.48, p = 0.000; hs-CRP: odds ratio 1.15, 95% confidence interval 1.04 to 2.03, p = 0.031; RDW: odds ratio 1.38, 95% confidence interval 1.11 to 2.97, p = 0.003; Table 3).

**Table 3:** Predictors of CAE in univariate and multivariate logistic regression analysis

**Variables**	**Univariate**	***P-*** **values**	**Multivariate**	***P-*** **values**
	**OR 95% CI**		**OR 95% CI**	
Age	1.04 1.01-1.07	0.017	0.94 0.63-1.37	0.331
Sex	1.15 1.04-1.19	0.004	1.13 1.13-1.25	0.006
Smoking	1.10 1.05-1.72	<0.001	1.22 1.08-2.48	0.000
WBC	1.02 1.05-1.47	0.042	1.05 0.39-1.17	0.608
PMC	1.07 1.00-155	0.050	0.87 0.57-1.35	0.219
Hs-CRP	1.13 1.08-2.38	0.005	1.15 1.04-2.03	0.031
RDW	1.38 1.12-2.07	0.002	1.38 1.11-1.97	0.003

CAE = coronary artery ectasia; OR = odds ratio; CI = confidence interval; WBC = white blood count; PMC = peripheral monocyte count; hs-CRP = high-sensitivity C-reactive protein; RDW = red blood cell distribution width.

### ROC analysis

**Figure 1:**
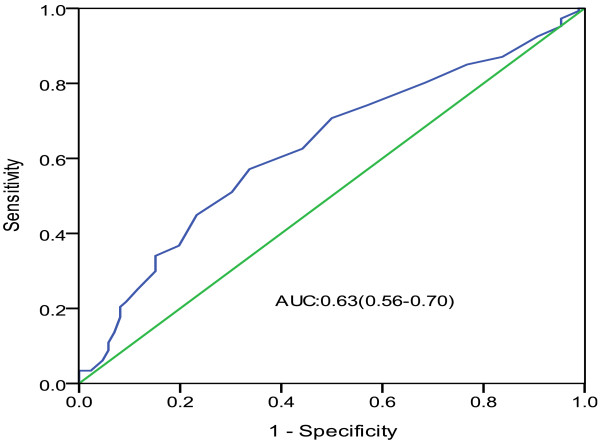
Receiver operating characteristic curve of red blood cell distribution width (RDW) for predicting the presence of isolated coronary artery ectasia (CAE) in studied patients (n = 113).

Finally, in a ROC curve analysis with area under curve (AUC) = 0.63, 95% CI: 0.56–0.70, we found that an RDW value of 12.73% was used as an effective cut-point in the segregation of the presence or absence of isolated CAE, a sensitivity of 57.7% and a specificity of 66.2% were obtained (Figure 1).

### Correlations of RDW with hs-CRP in patients with CAE

**Figure 2:**
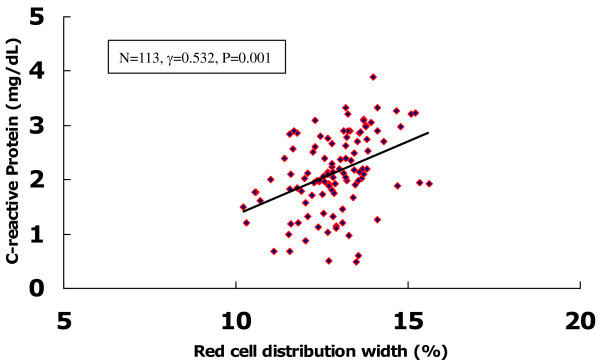
**Correlation analysis of plasma CRP levels and RDW in patients with isolated CAE.** There were marked positive correlation between the plasma levels of CRP and RDW (n = 113, γ=0.532, p = 0.001).

We also examined the correlation between the levels of RDW and hs-CRP in patients with CAE (n = 113). As shown in Figure 2, there was marked positive correlation between levels of RDW and hs-CRP in patients with isolated CAE (γ=0.532, p = 0.001).

## Discussion

The major findings of the present study are that (1) the RDW levels were significantly higher in patients with isolated CAE and CAD than that in angiographically normal controls; (2) the baseline RDW level was an independent predictor for the presence of isolated CAE; (3) there was a positive correlation between levels of RDW and CRP in patients with isolated CAE. These data, therefore, provided additional information regarding the association of RDW with isolated CAE in the cardiovascular field.

Although the clinical significance of isolated CAE is not fully understood, it has been demonstrated to be not a benign disorder and associated with a high risk of coronary events. Until now, the clinical studies on this unique inappropriate dilation of coronary arteries are relatively limited. Previous small sample studies suggested that CAE appeared to be associated with smoking and more male individuals [1,3,5]. Moreover, CAE has also been reported to be associated with atherosclerotic vascular disease, heterozygous familial hypercholesterolemia, usage of substances inhibitors including herbicide spray, acetylcholinesterase inhibitors and nitrate, prior arterial balloon angioplasty, polyarteritis nodosa and Kawasaki syndrome [1-5]. Therefore, the exploration of factors contributing to the development of isolated CAE or predictor biomarkers of isolated CAE may be of a great interesting.

RDW is a numerical measure of the variability in size of circulating erythrocytes. Generally, it is used, in combination with mean corpuscle volume, as an indicator of differential diagnosis of anemia [6]. Factors that contribute to increased erythrocyte size heterogeneity include iron or vitamin B12/folate deficiency, decreased erythrocyte life-span, impaired erythropoiesis and factors that contribute to erythrocyte fragmentation including increased fragility and destruction of red cell [6]. Recently, RDW has been shown to be a strong predictor for the presence of a variety of cardiovascular diseases and adverse outcomes in multiple settings [15]. Although those studies demonstrated the role of RDW in a variety of cardiovascular disease, the role of RDW in CAE, especially in association between RDW and isolated CAE are lacking. A recent small size study performed by Dogdu et al. [16] in 54 patients with CAE and 40 normal individuals reported that RDW were higher in the CAE patients compared with the control subjects. However, whether RDW is an independent predictor for patients with CAE was clearly not evaluated. Moreover, the potential mechanism was not also examined. In the present study we extended previous studies and for the first time found that baseline RDW levels is an independent predictor for patients with isolated CAE using univariate and multivariate logistic regression analysis.

The pathophysiologic mechanisms that the correlation of higher levels of RDW with CAE are largely unknown. It has been hypothesized that the systemic factors, such as inflammation and oxidative stress, might be the potential contributions. Previous studies suggested that higher RDW was associated with inflammatory markers such as soluble tumor necrosis factor receptors and CRP in the setting of atherosclerosis and other chronic diseases [17-19]. A strong association between RDW and inflammatory markers was also found in a large cohort of unselected adult outpatients, as well as patients with inflammatory bowel disease [20]. Pro-inflammatory cytokines may contribute to an increased RDW through enhanced oxidative stress [17]. Red blood cells have huge antioxidant capacity and serve as a primary oxidative sink, but they are prone to oxidative damage, which reduces cell survival and induces the release of juvenile erythrocytes into circulation [20,21]. Inflammation may also contributes to an increased RDW by impairing iron metabolism, inhibiting the production of or response to erythropoietin, and shortening red blood cell survival [22,23]. Additionally, inflammatory cytokines have been found to inhibit erythropoietin-induced erythrocyte maturation and induce the release of juvenile erythrocytes into circulation which lead to an increased RDW [24-26]. Interestingly, we have previously proposed that the inflammation may play a pathogenic role in isolated CAE [2]. This hypothesis has also been demonstrated by our clinical observations in patients with isolated CAE [4]. In the present study, the data further demonstrated a positive correlation between RDW and plasma CRP, suggesting an inflammatory mechanism may be involved in higher RDW levels in patients with CAE.

In conclusion, in the present study, our data demonstrated that RDW levels were higher, an independent predicting for isolated CAE, and positive correlated with inflammatory marker, CRP in patients with isolated CAE, suggesting that RDW, an easy, inexpensive, routinely reported test, whose assessment might allow the acquisition of significant diagnostic and prognosis information in patients with cardiovascular disorders [15] may be a useful marker and independent predictor for patients with isolated CAE. Further investigation is worthy of exploring the prognostic role of RDW in patients with isolated CAE.

### Study limitations

Although the association the increased RDW with the presence of isolated CAE has been established in the present study, whether RDW has a causal role contributing to isolated CAE or a marker of the disease process needs further investigation. Additionally, relative small sample size may limit the significance of the present study. Moreover, angiography cannot assess plaque burden, patients without evidence of luminal stenosis by angiography may also have plaque burden in the wall of the coronary arteries. It would be better to examine with intravascular techniques such as ultrasound whether the patients with isolated CAE had evidence of atherosclerotic plaque. Moreover, the relation of RDW level to the severity of isolated CAE or anatomical characteristics was not evaluated. The fact that the levels of folate, iron, and vitamin B12 were not evaluated was another limitation. Finally, the prognostic role of RDW in patients with isolated CAE needs to be investigated.

## Competing interests

The authors declare that they have no competing interests.

## Authors’ contributions

XLL collected and analyzed the data, drafted the manuscript. LFH participated in acquisition and analysis of data, and helped to draft the manuscript. YJJ, SPN, YLG, RXX, CGZ and LXJ collected and interpreted the data, and revised the manuscript. JJL conceived and designed this study, interpreted the data, and edited the manuscript. All authors read and approved the final manuscript.
